# The association between antibiotic use and outcomes of HCC patients treated with immune checkpoint inhibitors

**DOI:** 10.3389/fimmu.2022.956533

**Published:** 2022-08-17

**Authors:** Lilong Zhang, Chen Chen, Dongqi Chai, Chunlei Li, Yongjun Guan, Li Liu, Tianrui Kuang, Wenhong Deng, Weixing Wang

**Affiliations:** ^1^ Department of General Surgery, Renmin Hospital of Wuhan University, Wuhan, China; ^2^ Hubei Key Laboratory of Digestive System Disease, Wuhan, China

**Keywords:** immune checkpoint inhibitors, antibiotic, hepatocellular carcinoma, prognosis, meta-analysis

## Abstract

**Objective:**

Recently, immune checkpoint inhibitor (ICI) treatment has shown encouraging performance in improving the prognosis of hepatocellular carcinoma (HCC) patients. The gut microbiome plays a vital role in altering the efficacy of ICIs, which may be impacted by antibiotics. The aim of the meta-analysis is to estimate the influence of antibiotic use on the survival of HCC patients treated with ICIs.

**Methods:**

The literature review was conducted using databases like PubMed, EMBASE, Cochrane Library, CNKI, WANFANG DATA, VIP, Google Scholar, and ClinicalTrials.gov before May 15, 2022. The primary endpoints were overall survival (OS), progression-free survival (PFS), objective response rate (ORR), and disease control rate (DCR).

**Results:**

A total of six retrospective studies met the inclusion criteria. 1056 patients were included in the study, of which 352 (33.33%) received antibiotic treatment. The meta-analysis results revealed antibiotic use did not affect the OS (HR: 1.41, 95% CI: 0.96-2.08, *P* = 0.088) and PFS (HR: 1.21, 95% CI: 0.73-2.00, *P* = 0.459) in HCC patients treated with ICIs. Besides, the use of antibiotics did not reduce the ORR (OR: 1.06, 95% CI: 0.69-1.64, *P* = 0.784) and DCR (OR: 0.42, 95% CI: 0.09-2.06, *P* = 0.286) in HCC patients treated with ICIs.

**Conclusion:**

Current evidence reveals that antibiotic use does alter the therapeutic efficacy of ICIs in HCC patients.

**Systematic Review Registration:**

https://www.crd.york.ac.uk/, identifier CRD42022311948.

## Introduction

Primary liver cancer is the sixth most common type of cancer worldwide and ranks third in cancer-related deaths globally, amongst which hepatocellular carcinoma (HCC) accounts for 75-85% of all liver cancer cases (1). The prognosis of HCC is poor since most patients are diagnosed at an advanced stage or have limited liver reserve because of cirrhosis. This makes curative treatments, such as resection or ablative therapy, and liver transplantation difficult. In recent years, there has been significant development in advanced HCC therapeutics (2, 3); however, the effectiveness of systemic treatment like sorafenib is still suboptimal (4). Newer treatments, like multi-kinase inhibitors (regorafenib and cabozantinib) and monoclonal antibodies (ramucirumab), have a low overall survival (OS) of just 8.5-10.6 months (5).

Immune checkpoint inhibitors (ICIs), such as anti-programmed cell death 1 (anti-PD-1)/programmed cell death ligand 1 (PD-L1) and anti-cytotoxic T-lymphocyte-associated protein 4 (CTLA-4) antibodies, are novel and promising therapies that have been effective in prolonging survival in advanced HCC patients (6–9). The advanced HCC treated with nivolumab had an objective response rate (ORR) of 14% and a median OS of 15.1 months (6). More recently, the combination of nivolumab and ipilimumab has shown a manageable safety profile, with an ORR as high as 32% and durable responses (9). According to the promising results of early phase clinical trials, levatinib plus pembrolizumab is considered to have the potential to represent a novel treatment option for HCC patients (10). Despite the favorable outcomes, not all advanced HCC patients respond to ICI treatment. Patients who do not respond to ICI therapy often experience tumor progression and may even suffer severe immune adverse effects, such as pneumonia, myocarditis, and hepatitis, all of which can be fatal (11, 12). Therefore, the search for potential factors influencing its efficacy is extremely necessary for a more targeted selection of treatment populations in clinical practice (13).

In 2015, a heavyweight study correlating the effect of intestinal microbiomes on the efficacy of ICI treatment was published for the first time in Science (14). Next, it has been reported that intestinal microbiomes can influence the anti-PD-1 treatment response in HCC patients, with responders having higher taxonomic richness and more gene counts than non-responders (15, 16). It is well known that antibiotics are the most common clinical cause of alterations in gut flora. Recently, two studies published in the same issue of “liver cancer” have come to opposite conclusions regarding the effect of antibiotics on the efficacy of ICI treatment in HCC (17, 18). Besides, some similar studies have been undertaken throughout the world, but there has been no consensus established. To address these clinical problems, the first meta-analysis was conducted by our team to investigate whether the antibiotics influence the efficacy of ICI therapy in HCC patients. This will provide evidence-based results for the clinical application of antibiotics in HCC patients undergoing ICI treatment, thereby maximizing the clinical benefit for patients.

## Methods

### Literature search strategies

The Preferred Reporting Items for Systematic Reviews and Meta-Analyses (PRISMA) guidelines were strictly followed while conducting the meta-analysis (19). The protocol for the meta-analysis is available on PROSPERO (CRD42022311948). The literature review was conducted using databases like PubMed (https://pubmed.ncbi.nlm.nih.gov/), EMBASE (https://www.embase.com/), Cochrane Library (https://www.cochranelibrary.com/), CNKI (https://www.cnki.net/), WANFANG DATA (https://www.wanfangdata.com.cn/), and VIP (http://www.cqvip.com/) before May 15, 2022. “Anti-Bacterial Agents”[Mesh], “Immune Checkpoint Inhibitors”[Mesh], “Liver Neoplasms”[Mesh], “Carcinoma, Hepatocellular “[Mesh], and their entry terms were searched in [All Fields]. Detailed search strategies are presented in **Table S1**. A gray literature search was performed using Google Scholar to find reports that were not indexed in the previously mentioned databases, such as conference abstracts, presentations, and unpublished trial data. The Clinical Trial Registration Platform, like ClinicalTrials.gov (https://clinicaltrials.gov/), was utilized to search for ongoing trials. Besides, we also manually searched the reference lists of eligible papers.

### Study selection criteria

Full-text articles and conference abstracts were included based on the inclusion criteria, which are as follows: (1) patients diagnosed with HCC; (2) patients treated with ICIs; (3) patients divided into the non-antibiotic group and antibiotic group based on the history of antibiotic use; (4) provided at least one of the outcomes of interest [OS, progression-free survival (PFS), ORR, and disease control rate (DCR)]. Articles that failed to report information about subjects, such as sample size and other basic information, were discarded. When studies reported overlapping patient populations, only the article with the most complete data and rigorous methodology was selected.

### Data extraction and quality assessment

Data extraction mainly focused on the author, publication year, study type, period, and region, the number of patients, types of ICI treatment, treatment-related outcomes (OS, PFS, ORR, and DCR), covariates of multivariate analysis for OS and PFS, the reason for antibiotic use, types and timing of antibiotic use, and antibiotic (median) duration. If both univariate and multivariate analyses were used to calculate the hazard ratio (HR), the latter was preferred because the result was adjusted for confounding factors and was more accurate. Authors were contacted if the relevant data was not immediately accessible from published abstracts or articles. The Newcastle-Ottawa Scale (NOS) score was applied to estimate the quality of the selected literature 42. Literature with a score ≥ 7 was regarded as high-quality ones. All the above steps were independently cross-checked by two authors (Zhang Lilong and Chen Chen), and all differences were addressed by the senior author (Deng Wenhong and Wang Weixing).

### Statistical methods

Statistical analysis was conducted using Stata SE15.0. The relationship between the efficacy of ICI therapy and antibiotic usage was reported as an odds ratio (OR) with a 95% confidence interval (95% CI). The effect of antibiotic use on the risk of survival in HCC patients was calculated using the HR and 95% CI. The chi-square test was applied to determine the statistical heterogeneity among the studies. *P* > 0.1 and I^2^ < 50% revealed low heterogeneity where a fixed-effect model was adopted; otherwise, the random-effect model was utilized. The subgroup analysis was carried out to minimize the impact of heterogeneity on the meta-analysis. Publication bias was measured using Begg’s and Egger’s tests. Sensitivity analysis by the leave-one-out method was used to assess the stability of the results. All *P* values were two-sided, and the statistical significance was set at *P* < 0.05.

## Results

### Studies retrieved and characteristics

**Figure 1:**
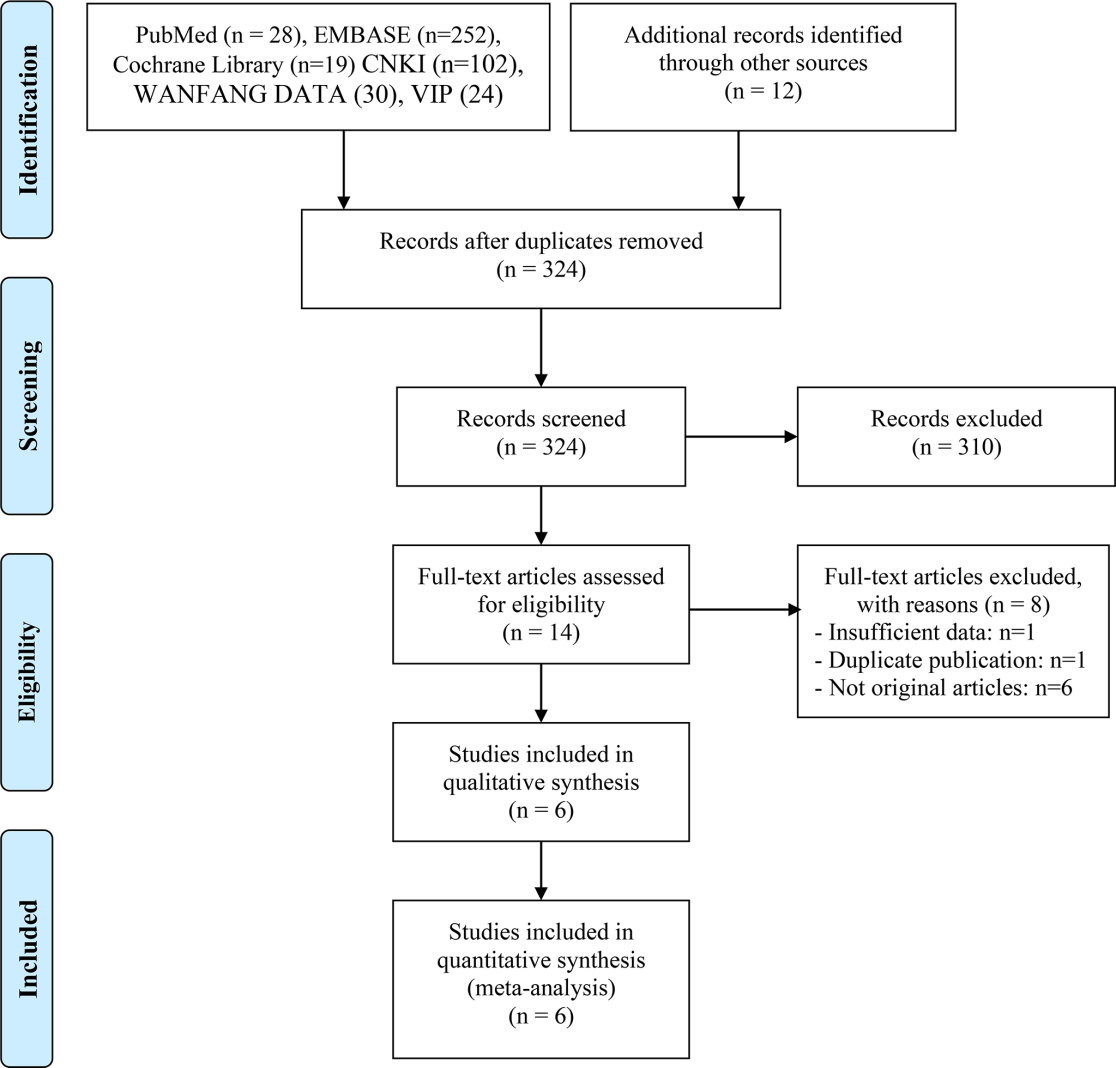
The flow diagram of identifying eligible studies.

467 eligible records were screened for their titles and abstracts to check for their eligibility. After a detailed analysis of 14 full-text records, we found that six studies met the inclusion criteria (17, 18, 20–23). The articles by Ren *et al.* (24) and Jun *et al.* (25) were excluded from the meta-analysis due to insufficient data and duplicate publication, respectively. The flow diagram of identifying eligible studies is shown in **Figure 1**. The baseline characteristics and the scores of the quality assessment are shown in **Table 1**. The score for four articles was 7 or 8 points, and the articles were deemed high-quality. The remaining two studies received 5 and 6 points and were deemed moderate-quality. The information from the survival analysis and antibiotic use was also listed in **Table S2**.

**Table 1:** Baseline characteristics of included studies.

Author	Year	Study type	Study period	Study region	Number of antibiotic	Number of non- antibiotic	Types of ICI treatment	Outcomes	Quality (NOS Score)
Fessas et al.	2021	Retrospective	2017-2019	Europe, America, Asia	170	279	Anti-PD(L)-1 and/or Anti-CTLA-4	OS, PFS, ORR, DCB	8
Spahn et al.	2020	Retrospective	08/2015-12/2019	Germany, Austria, Switzerland	21	78	Anti-PD(L)-1 (nivolumab, pembrolizumab)	OS, PFS	7
Cheung et al.	2021	Retrospective	01/2014-12/2019	China(Hong Kong)	109	286	Anti-PD(L)-1 (nivolumab, pembrolizumab) and/or CTLA-4 (ipilimumab)	OS	7
Alshammari et al.	2021	Retrospective	–	Saudi Arabia	20	39	Anti-PD(L)-1 (nivolumab)	OS, ORR	5
Chen_xj et al.	2021	Retrospective	09/2018-06/2020	Chian(Nanchang)	18	22	Anti-PD(L)-1 (camrelizumab, tislelizumab, sintilimab, toriplimab, pembrolizumab)	OS, PFS, ORR, DCB	7
Chen_q et al.	2018	Retrospective	05/2016-12/2017	Chian(Fujian)	14	19	Anti-PD(L)-1 (nivolumab, pembrolumab)	OS, PFS, ORR, DCB	6

ICI, Immune checkpoint inhibitors; PD-1, programmed cell death 1; PDL-1, programmed cell death ligand 1; CTLA4, cytotoxic T-lymphocyte-associated protein 4; OS, overall survival; PFS, progression-free survival; ORR, objective response rate; DCR, disease control rate; NOS, Newcastle-Ottawa Scale.

### Overall survival

**Figure 2:**
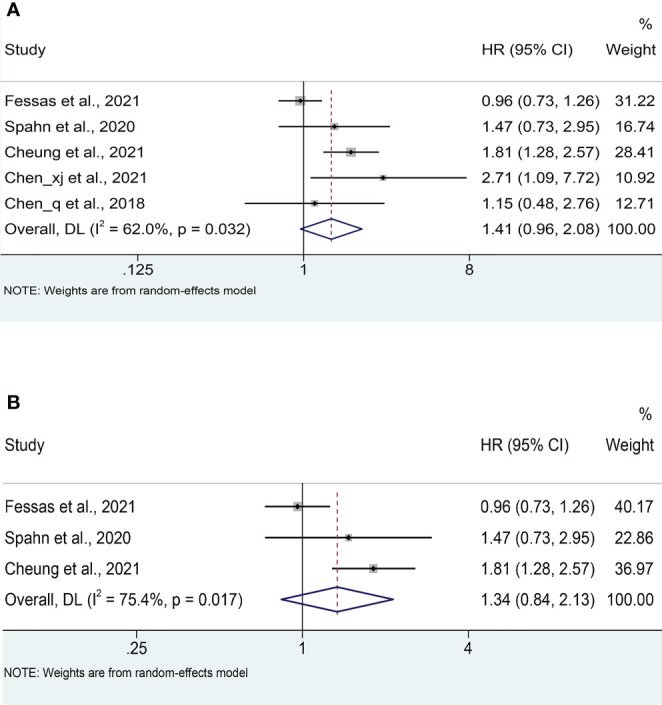
**(A)** Meta-analysis of the overall OS. **(B)** Meta-analysis of OS after excluding Chinese literature. OS: overall survival; HR: hazard ratio; CI: confidence interval.

**Figure 3:**
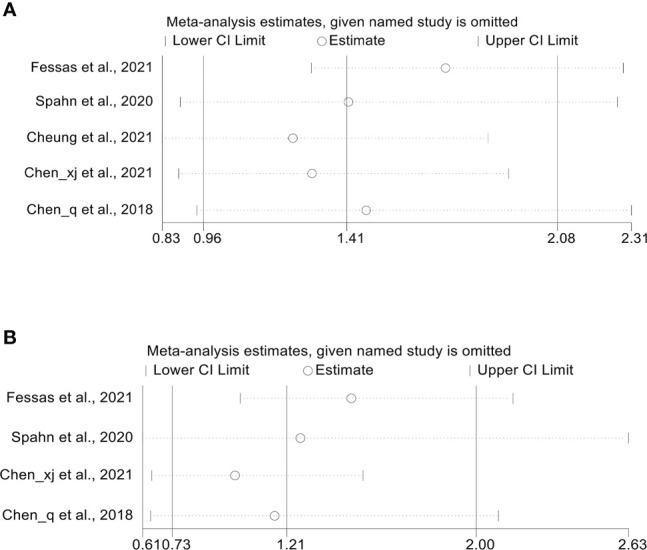
**(A)** Sensitivity analysis of the overall OS. **(B)** Sensitivity analysis of the overall PFS. OS, overall survival; PFS, progression-free survival; CI, confidence interval.

Five studies (18, 20–23) reported median OS, with a mean median OS of 8.275 months, ranging from 3.3 to 15.3 months in the antibiotic group; while the mean median OS in the non-antibiotic group was 12.62 months, ranging from 4 to 17.4 months (**Figure S1A**). The meta-analysis for OS was performed using survival data from 5 studies (17, 18, 21–23), which included 1016 participants (332 with antibiotics versus 684 with non-antibiotics). As illustrated in **Figure 2A**, significant heterogeneity was observed in the studies (I^2^ = 62.0%, *P* = 0.032), therefore a random-effects model was applied. The results revealed that antibiotic use did not shorten OS in HCC patients treated with ICIs (HR: 1.41, 95% CI: 0.96-2.08, *P* = 0.088). Begg’s and Egger’s tests indicated no significant publication bias (Begg’s test *P* = 0.906; Egger’s test *P* = 0.940). To estimate the influence of each study on the overall meta-analysis, we conducted a sensitivity analysis using the leave-one-out method. The results showed that no single study significantly impacted the pooled HR of OS (**Figure 3A**). Besides, two Chinese articles were excluded from the analysis to investigate the reliability of the results further. As shown in **Figure 2B**, excluding the two articles did not change the results (HR: 1.34, 95% CI: 0.84-2.13, *P* = 0.222). Thus, it would be safe to assume that the meta-analysis results were relatively stable and reliable.

### Progression-free survival

**Figure 4:**
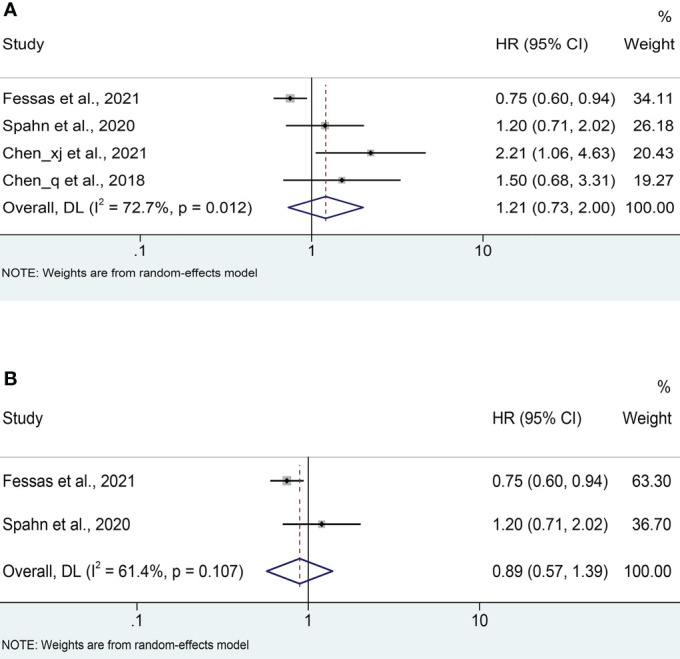
**(A)** Meta-analysis of the overall PFS. **(B)** Meta-analysis of PFS after excluding Chinese literature. PFS, progression-free survival; HR, hazard ratio; CI, confidence interval.

Four cohort studies (18, 21–23) reported median PFS with a mean of 4.95 months ranging from 2 to 6.7 months in the antibiotic group and 5.975 months ranging from 3.7 to 8.9 months in the non-antibiotic group (**Figure S1B**). Pooled data from 4 studies (18, 21–23) with a total of 621 patients (223 with antibiotics versus 398 with non-antibiotics) was used to assess the association between antibiotic usage and PFS. Due to significant heterogeneity, a random-effects model was used (I^2^ = 72.7%, *P* = 0.012). The results revealed no effect of the use of antibiotics on the PFS of HCC patients (HR: 1.21, 95% CI: 0.73-2.00, *P* = 0.459, **Figure 4A**). Begg’s test shows no publication bias in the results (*P* = 0.308), while Egger’s test shows publication bias (*P* = 0.034). Therefore, the “trim and fill” method was further used to verify the effect of publication bias on the meta-analysis results. We found that the trend of PFS remained unchanged following the correction by the “trim and fill” method. The sensitivity analysis results also confirmed that no single study could substantially affect the pooled HR of PFS (**Figure 3B**). After excluding the two Chinese articles, the results were still consistent (**Figure 4B**, I^2^ = 61.4%, *P* = 0.107; HR: 0.89, 95% CI: 0.57-1.39, *P* = 0.608), thereby confirming the reliability of our conclusion.

### Objective response rate and disease control rate

**Figure 5:**
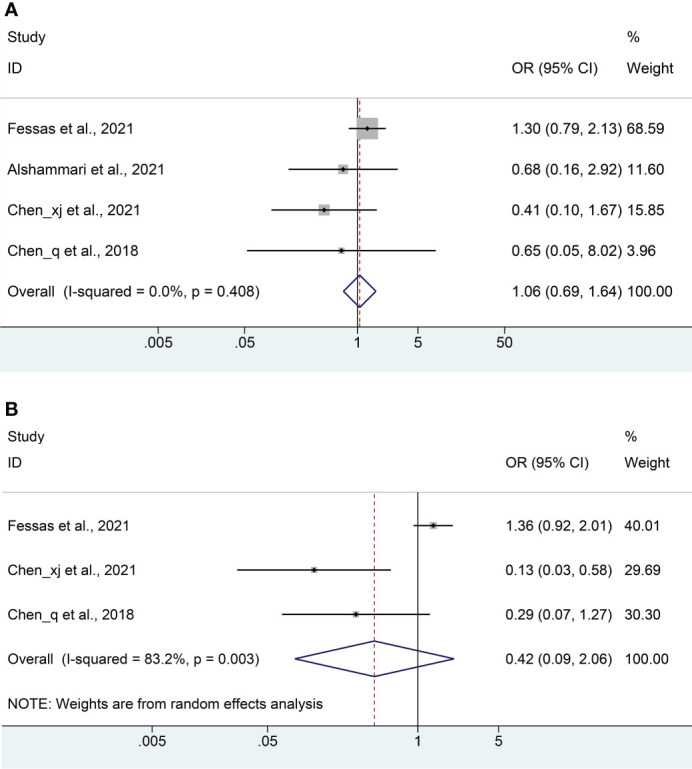
Meta-analysis of the objective response rate **(A)** and disease control rate **(B)**. OR, odds ratio; CI, confidence interval.

Four studies (18, 20–22) with a total of 581 patients (222 with antibiotics versus 359 with non-antibiotics) were included in the meta-analysis of ORR. No significant heterogeneity was included in the studies (I^2^ = 0%, *P* = 0.408), and a fix-effects model was applied. We found that antibiotic usage did not reduce ORR in HCC patients treated with ICIs (OR: 1.06, 95% CI: 0.69-1.64, *P* = 0.784, **Figure 5A**). No remarkable publication biases were observed using the Begg’s (*P* = 1.000) and Egger’s tests (*P* = 0.153).

The DCR meta-analysis included 3 cohort studies (18, 21, 22) with a total of 522 patients (202 with antibiotics versus 320 with non-antibiotics). Since significant heterogeneity was observed in the included studies (I^2^ = 82.3%, *P* = 0.003), a random-effects model was performed. The results revealed no significant difference in DCR between antibiotic and non-antibiotic groups (OR: 0.42, 95% CI: 0.09-2.06, *P* = 0.286, **Figure 5B**). Similarly, no remarkable publication biases were observed using the Begg’s (*P* = 0.296) and Egger’s tests (*P* = 0.126).

In addition, we also found that the use of antibiotics did not affect the complete response rate (**Figure S2A**, I^2^ = 0.0%, *P* = 0.968; OR: 0.406, 95% CI: 0.040-4.089, *P* = 0.444) and partial response rate (**Figure S2B**, I^2^ = 0.0%, *P* = 0.532; OR: 0.610, 95% CI: 0.172-2.162, *P* = 0.444) in the HCC patients with ICI therapy.

## Discussion

With the increased use of ICIs in cancer therapeutics, tremendous effort has been made to uncover possible factors that impact its efficacy. Among the identified factors, a growing body of evidence has indicated a crucial role for the intestinal microbiome (26). Frequent use of antibiotics interferes with intestinal flora, and its effect on the efficacy of ICIs has recently sparked an intense debate (27). Currently, several meta-analyses have revealed that antibiotic administration may be related to poor prognosis in tumor patients receiving ICIs (28–31). However, these studies are focused on lung, melanoma, bladder, and kidney cancers, and no studies on liver cancer have been conducted. To the best of our knowledge, this is the first meta-analysis that investigated the relationship between antibiotics and ICI efficacy in the treatment of HCC. We present all the available evidence to confirm that antibiotic use does not impact the prognosis and response in HCC patients treated with ICIs. Publication bias and sensitivity analyses further confirmed the dependability of our results.

It has been established that in patients with chronic liver disease, intestinal barrier dysfunction allows for increased intestinal bacterial translocation (32). Long-term exposure to lipopolysaccharide from intestinal microbiomes is crucial to the development of cirrhosis and HCC by activating the TGF-β pathway, which is an important molecular driver for anti-apoptotic and proliferative signaling in hepatocyte (33, 34). Once HCC is established, the gut-liver axis keeps up to affect the anti-tumor immune response and perturbation of the intestinal bacteria, in which antibiotics have a direct impact on the tumor microenvironment (35). The general deleterious effect on the outcomes of antibiotic use in malignancy is considered to be related to the detrimental impact of antibiotics on decreasing the diversity and taxonomy of the gut microbiome, causing a reduction in *Bifidobacteria*, *Ruminococcus*, and *Akkermansia*, while favoring the growth of other specific bacteria, such as *Bacteroides*  (16, 36). Such bacteria can induce immunosuppression by promoting myeloid-derived suppressor cells, FOXP3^+^ and CD4^+^ CD25^+^ T-regulatory (Treg) cells, and by producing prostaglandins, which negatively correlate with the ICI response (37).

HCC differs from other cancers in that it develops in the context of cirrhosis, a pathological state already linked to immunosuppressive microbiota (38). Cirrhosis is accompanied by a dysbiosis of intestinal microbiota, with an increase in immunosuppressive bacteria and a decrease in beneficial bacteria. Studies in mouse models show a decrease in *Bifidobacterium* and an increase in gram-negative bacteria, such as *Bacteroides* and *Escherichia Coli*, presumably contributing to the progression of HCC (39). Thus, disrupting this immunosuppressive interaction by antibiotics may be a plausible explanation for the no effect of antibiotics on the poor prognosis of HCC patients treated with ICIs. Unlike lung and melanoma cancers, HCC has a distinct immunosuppressive tumor microenvironment (40), due to the abundant recruitment of myeloid suppressor cells and macrophages, which directly suppress cytotoxic T cells and produce chemokines, such as CCL17, CCL18, and CCL22, which further attract Treg cells. Interestingly, Han *et al.* recently demonstrated that antibiotic-induced microbiota dysbiosis enhances the anti-tumor efficacy of gamma delta T cells during immunotherapy in a mouse model (41). To sum up, the impact of antibiotics on ICI therapy in HCC patients is inconclusive. In the comprehensive meta-analysis of six studies, we found that antibiotic use had no effect on the outcomes of ICI treatment in patients with HCC.

Notably, some inherent limitations do exist in this study. To begin with, we present a meta-analysis that depends on the published articles. The lack of enough data prevented us from conducting subgroup analyses based on the type of antibiotic used, route of administration, duration of use, etc. In addition, the study included mainly Asians, and the total number of patients analyzed was relatively small. Finally, we were unable to examine the association between antibiotic use and ICI-induced adverse events, which should be highlighted in our follow-up work. Therefore, future larger, multi-institutional studies with standardized prospective data collection are needed to further confirm our findings above.

## Conclusion

Current evidence reveals that, unlike other oncological indications, antibiotic use does not affect the efficacy of ICI treatment in HCC patients.

## Data availability statement

The original contributions presented in the study are included in the article/**Supplementary Material**. Further inquiries can be directed to the corresponding authors.

## Author contributions

LZ, CC, WD, and WW conceived and designed the study. LZ, DC, CC, YG, LL, and CL were responsible for the collection and assembly of data, data analysis, and interpretation. LZ, DC, and CC were involved in writing the manuscript. LZ, CC, TK, and WW revised the manuscript. All the work was performed under WD and WW instruction. All authors contributed to the article and approved the submitted version.

## Funding

This work was supported by grants from the Natural Science Foundation of China (No. 82172855, 81870442) and the Natural Science Foundation of Hubei Province, China (No. 220171530).

## Conflict of interest

The authors declare that the research was conducted in the absence of any commercial or financial relationships that could be construed as a potential conflict of interest.

## Publisher’s note

All claims expressed in this article are solely those of the authors and do not necessarily represent those of their affiliated organizations, or those of the publisher, the editors and the reviewers. Any product that may be evaluated in this article, or claim that may be made by its manufacturer, is not guaranteed or endorsed by the publisher.

## Supplementary material

Supplementary Figure 1 The median overall survival (A) and median progression-free survival (B) for included studies.

Supplementary Figure 2 Meta-analysis of the complete response rate (A) and partial response rate (B). OR: odds ratio; CI: confidence interval
