# Supplementary figures and images for: The association between antibiotic use and outcomes of HCC patients treated with immune checkpoint inhibitors
Source: Front Immunol. 2022 Aug 17;13:956533. doi: 10.3389/fimmu.2022.956533 (PMC9429218; doi:10.3389/fimmu.2022.956533)

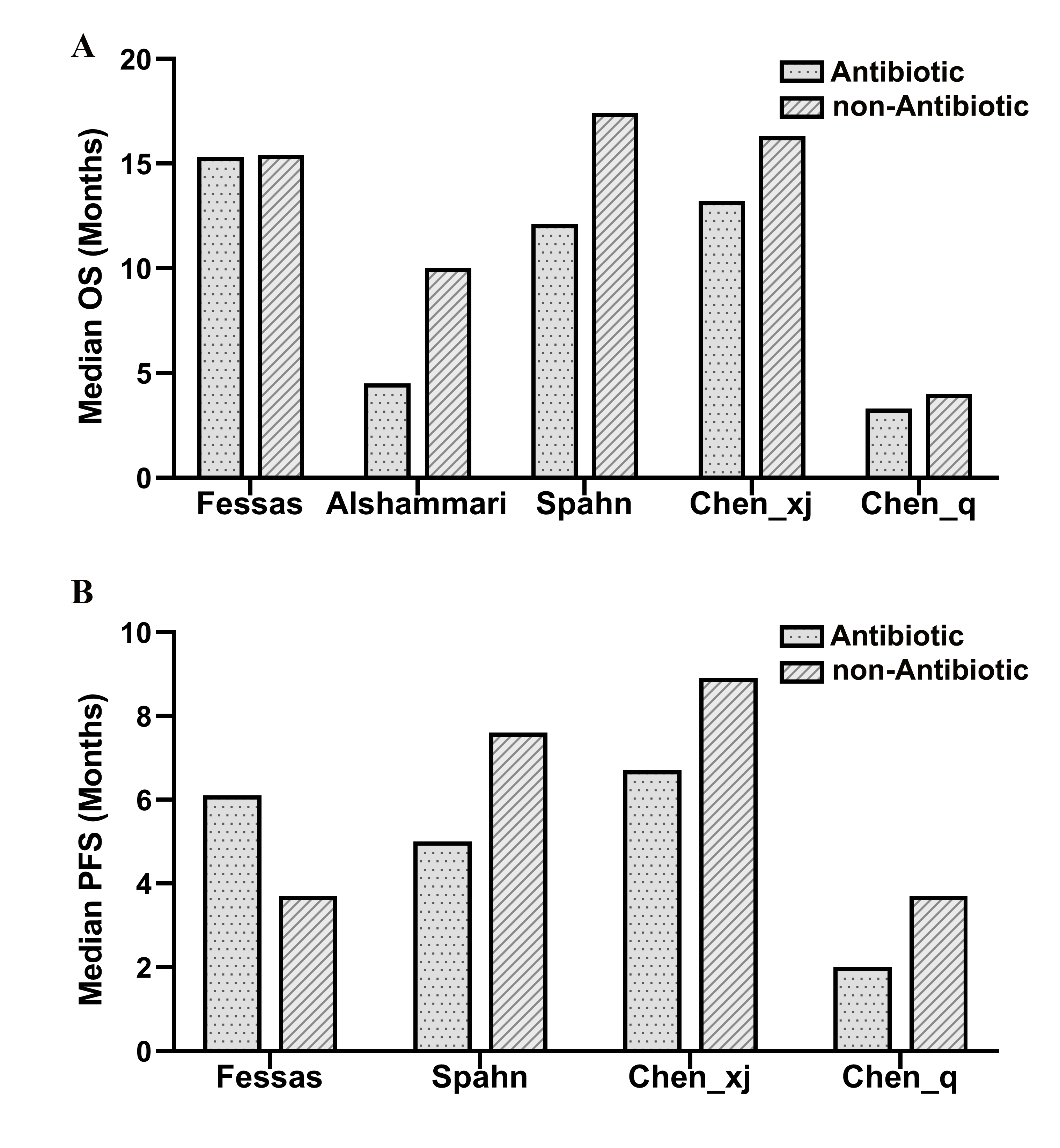

Supplement: Supplementary Figure 1 — The median overall survival (A) and median progression-free survival (B) for included studies. [file Image_1.tif]

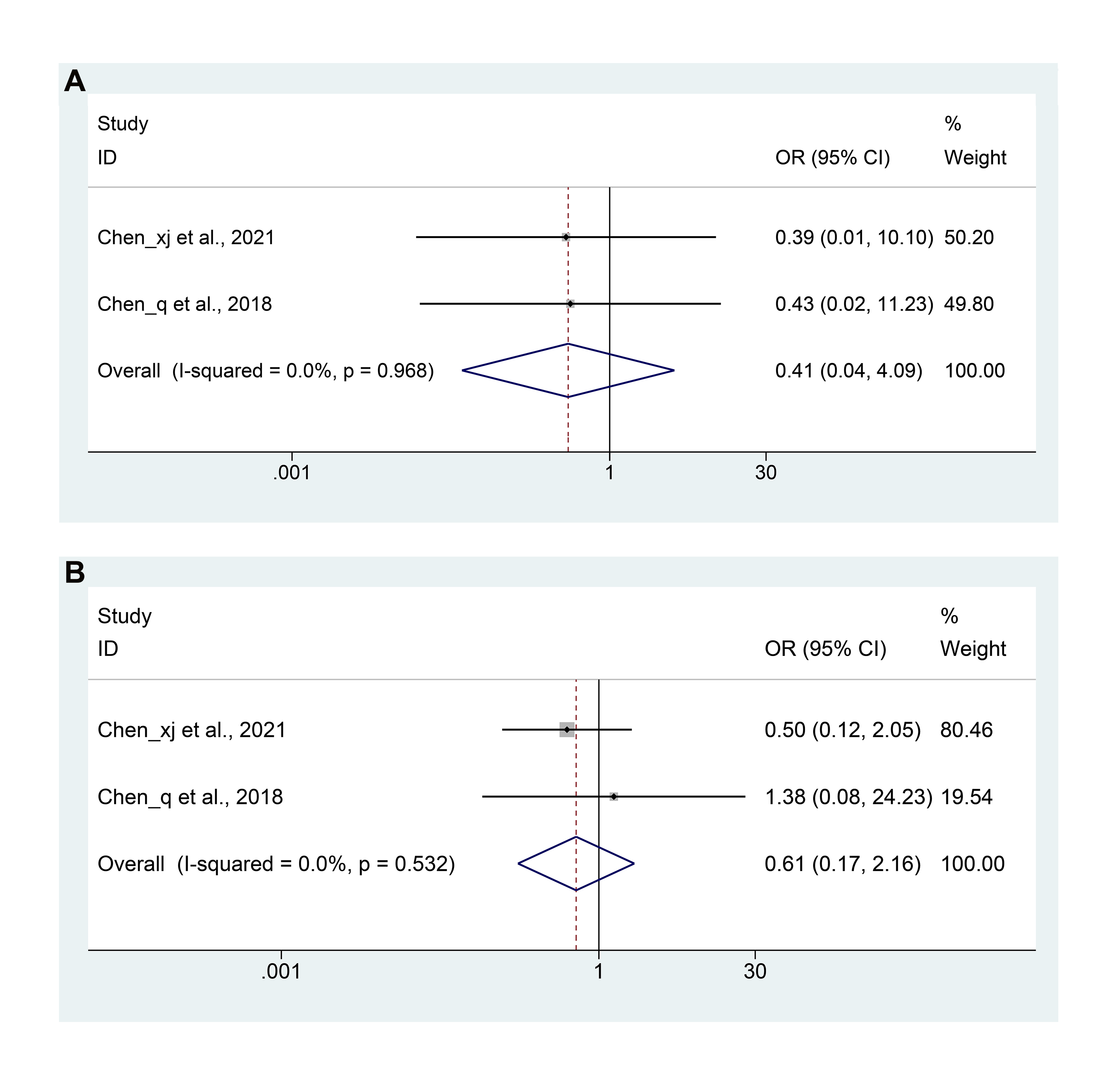

Supplement: Supplementary Figure 2 — Meta-analysis of the complete response rate (A) and partial response rate (B). OR: odds ratio; CI: confidence interval [file Image_2.tif]
