# Supplementary material for: The association between antibiotic use and outcomes of HCC patients treated with immune checkpoint inhibitors
Source: Front Immunol. 2022 Aug 17;13:956533. doi: 10.3389/fimmu.2022.956533 (PMC9429218; doi:10.3389/fimmu.2022.956533)
Supplement: Supplementary file 3 [file Table_1.docx]

1. **The search strategy of Pubmed**

(((((((((((((((((((((((((Agents, Anti-Bacterial) OR (Anti Bacterial Agents)) OR (Antibacterial Agents)) OR (Antibacterial Agent)) OR (Anti-Bacterial Compounds)) OR (Anti Bacterial Compounds)) OR (Anti-Bacterial Agent)) OR (Anti Bacterial Agent)) OR (Anti-Bacterial Compound)) OR (Anti Bacterial Compound)) OR (Bacteriocidal Agents)) OR (Bacteriocidal Agent)) OR (Bacteriocide)) OR (Bacteriocides)) OR (Anti-Mycobacterial Agents)) OR (Anti Mycobacterial Agents)) OR (Anti-Mycobacterial Agent)) OR (Anti Mycobacterial Agent)) OR (Antimycobacterial Agent)) OR (Antimycobacterial Agents)) OR (Agents, Antimycobacterial)) OR (Antibiotics)) OR (Antibiotic)) OR ("Anti-Bacterial Agents"[Mesh])) AND (((((((((((((((((((((((((((((Checkpoint Inhibitors, Immune) OR (Immune Checkpoint Inhibitor)) OR (Immune Checkpoint Blockers)) OR (Immune Checkpoint Blockade)) OR (Immune Checkpoint Inhibition)) OR (PD-L1 Inhibitors)) OR (PD L1 Inhibitors)) OR (PD-L1 Inhibitor)) OR (PD L1 Inhibitor)) OR (Programmed Death-Ligand 1 Inhibitors)) OR (Programmed Death Ligand 1 Inhibitors)) OR (PD-1-PD-L1 Blockade)) OR (PD 1 PD L1 Blockade)) OR (CTLA-4 Inhibitors)) OR (CTLA 4 Inhibitors)) OR (CTLA-4 Inhibitor)) OR (CTLA 4 Inhibitor)) OR (Cytotoxic T-Lymphocyte-Associated Protein 4 Inhibitors)) OR (Cytotoxic T Lymphocyte Associated Protein 4 Inhibitors)) OR (Cytotoxic T-Lymphocyte-Associated Protein 4 Inhibitor)) OR (Cytotoxic T Lymphocyte Associated Protein 4 Inhibitor)) OR (PD-1 Inhibitors)) OR (PD 1 Inhibitors)) OR (PD-1 Inhibitor)) OR (Inhibitor, PD-1)) OR (PD 1 Inhibitor)) OR (Programmed Cell Death Protein 1 Inhibitor)) OR (Programmed Cell Death Protein 1 Inhibitors)) OR ("Immune Checkpoint Inhibitors"[Mesh]))) AND ((((((((((Hepatocellular Carcinomas) OR (Adult Liver Cancer)) OR (Adult Liver Cancers)) OR (Liver Cell Carcinoma)) OR (Liver Cell Carcinomas)) OR (Hepatocellular Carcinoma)) OR (Hepatoma)) OR (Hepatomas)) OR ("Carcinoma, Hepatocellular"[Mesh]) ) OR (("Liver Neoplasms"[Mesh]) OR ((((((((((((((Neoplasms, Hepatic) OR (Neoplasms, Liver)) OR (Liver Neoplasm)) OR (Hepatic Neoplasms)) OR (Hepatic Neoplasm)) OR (Cancer of Liver)) OR (Hepatocellular Cancer)) OR (Hepatocellular Cancers)) OR (Hepatic Cancer)) OR (Hepatic Cancers)) OR (Liver Cancer)) OR (Liver Cancers)) OR (Cancer of the Liver)) OR (Cancer, Hepatocellular))))

1. **The search strategy of Cochrane**

#1 MeSH descriptor: [Anti-Bacterial Agents] explode all trees

#2 (Agents, Anti-Bacterial) OR (Anti Bacterial Agents) OR (Antibacterial Agents) OR (Antibacterial Agent) OR (Anti-Bacterial Compounds)

#3 (Anti Bacterial Compounds) OR (Anti-Bacterial Agent) OR (Anti Bacterial Agent) OR (Anti-Bacterial Compound) OR (Anti Bacterial Compound)

#4 (Bacteriocidal Agents) OR (Bacteriocidal Agent) OR (Bacteriocide) OR (Bacteriocides) OR (Anti-Mycobacterial Agents)

#5 (Anti Mycobacterial Agents) OR (Anti-Mycobacterial Agent) OR (Anti Mycobacterial Agent) OR (Antimycobacterial Agent) OR (Antimycobacterial Agents)

#6 (Agents, Antimycobacterial) OR (Antibiotics) OR (Antibiotic)

#7 #1 OR #2 OR #3 OR #4 OR #5 OR #6

#8 MeSH descriptor: [Immune Checkpoint Inhibitors] explode all trees

#9 (Checkpoint Inhibitors, Immune) OR (Immune Checkpoint Inhibitor) OR (Immune Checkpoint Blockers) OR (Immune Checkpoint Blockade) OR (Immune Checkpoint Inhibition)

#10 (PD-L1 Inhibitors) OR (PD L1 Inhibitors) OR (PD-L1 Inhibitor) OR (PD L1 Inhibitor) OR (Programmed Death-Ligand 1 Inhibitors)

#11 (Programmed Death Ligand 1 Inhibitors) OR (CTLA-4 Inhibitors) OR (CTLA 4 Inhibitors)

#12 (CTLA-4 Inhibitor) OR (CTLA 4 Inhibitor) OR (Cytotoxic T-Lymphocyte-Associated Protein 4 Inhibitors) OR (Cytotoxic T Lymphocyte Associated Protein 4 Inhibitors) OR (Cytotoxic T-Lymphocyte-Associated Protein 4 Inhibitor)

#13 (Cytotoxic T Lymphocyte Associated Protein 4 Inhibitor) OR (PD-1 Inhibitor) OR (Inhibitor, PD-1) OR (PD 1 Inhibitor) OR (Programmed Cell Death Protein 1 Inhibitor)

#14 (PD-1 Inhibitors) OR (PD 1 Inhibitors) OR (Programmed Cell Death Protein 1 Inhibitors)

#15 #8 OR #9 OR #10 OR #11 OR #12 OR #13 OR #14

#16 MeSH descriptor: [Carcinoma, Hepatocellular] explode all trees

#17 MeSH descriptor: [Liver Neoplasms] explode all trees

#18 (Hepatocellular Carcinomas) OR (Adult Liver Cancer) OR (Adult Liver Cancers) OR (Liver Cell Carcinoma) OR (Liver Cell Carcinomas)

#19 (Hepatocellular Carcinoma) OR (Hepatoma) OR (Hepatomas) OR (Neoplasms, Hepatic) OR (Neoplasms, Liver)

#20 (Liver Neoplasm) OR (Hepatic Neoplasms) OR (Hepatic Neoplasm) OR (Cancer of Liver) OR (Hepatocellular Cancer)

#21 (Hepatocellular Cancers) OR (Hepatic Cancer) OR (Hepatic Cancers) OR (Liver Cancers) OR (Cancer of the Liver)

#22 (Cancer, Hepatocellular)

#23 #16 OR #17 OR #18 OR #19 OR #20 OR #21 OR #22

#24 #7 AND #15 AND #23

1. **The search strategy of Embase**

#1 carcinoma, AND hepatic AND cell OR (carcinoma, AND hepatocellular) OR (carcinoma, AND liver) OR (carcinoma, AND liver AND cell) OR (hepatic AND carcinoma) OR (hepatic AND cell AND carcinoma) OR hepatocarcinoma OR (hepatocellular AND carcinoma) OR (liver AND carcinoma) OR hepatoma OR (liver AND carcinoma AND rupture) OR (malignant AND hepatoma) OR (primary AND liver AND carcinoma) OR (cancer, AND liver) OR (hepatic AND cancer) OR (liver AND primary AND cancer) OR (primary AND liver AND cancer)

#2 'liver cell carcinoma'/mj

#3 'liver cancer'/mj

#4 #1 OR #2 OR #3

#5 antibiotic OR antibiotics OR (anti AND bacterial AND agent) OR (anti AND bacterial AND agents) OR (anti AND infective AND agents) OR (anti AND infectives, AND otic) OR ('anti bacterial' AND agents) OR ('anti infective' AND agents) OR ('anti infectives,' AND otic) OR antibacterial OR (antiinfectives, AND otic) OR antimicrobial OR antiseptic OR (chemotherapeutic AND agent) OR (chemotherapeutic AND drug) OR chemotherapeutica OR (microbiological AND agent)

#6 'antiinfective agent'/mj

#7 'antibiotic agent'/mj

#8 #5 OR #6 OR #7

#9 checkpoint AND inhibitors, AND immune OR (immune AND checkpoint AND inhibitor) OR (immune AND checkpoint AND blockers) OR (immune AND checkpoint AND blockade) OR (immune AND checkpoint AND inhibition) OR ('pd l1' AND inhibitors) OR (pd AND l1 AND inhibitors) OR ('pd l1' AND inhibitor) OR (pd AND l1 AND inhibitor) OR (programmed AND 'death ligand' AND 1 AND inhibitors) OR (programmed AND death AND ligand AND 1 AND inhibitors) OR ('ctla 4' AND inhibitors) OR (ctla AND 4 AND inhibitors) OR ('ctla 4' AND inhibitor) OR (ctla AND 4 AND inhibitor) OR (cytotoxic AND 't lymphocyte associated' AND protein AND 4 AND inhibitors) OR (cytotoxic AND t AND lymphocyte AND associated AND protein AND 4 AND inhibitors) OR (cytotoxic AND 't lymphocyte associated' AND protein AND 4 AND inhibitor) OR (cytotoxic AND t AND lymphocyte AND associated AND protein AND 4 AND inhibitor) OR (inhibitor, AND 'pd 1') OR ('pd 1' AND inhibitor) OR (pd AND 1 AND inhibitor) OR (programmed AND cell AND death AND protein AND 1 AND inhibitor) OR ('pd 1' AND inhibitors) OR (pd AND 1 AND inhibitors) OR (programmed AND cell AND death AND protein AND 1 AND inhibitors)

#10 'immune checkpoint inhibitor'/mj

#11 #9 OR #10

#12 #4 AND #8 AND #11
