# Supplementary material for: The association between antibiotic use and outcomes of HCC patients treated with immune checkpoint inhibitors
Source: Front Immunol. 2022 Aug 17;13:956533. doi: 10.3389/fimmu.2022.956533 (PMC9429218; doi:10.3389/fimmu.2022.956533)
Supplement: Supplementary file 4 [file Table_2.docx]

**Table S2** The baseline information about the survival analysis and antibiotics use in included studies

| Author | Covariates of multivariate analysis for OS and PFS | Reason for antibiotics | Types of antibiotics | Timing of antibiotics  use | Antibiotics (median) duration (days) |
| --- | --- | --- | --- | --- | --- |
| Fessas et al. | Gender, age, viral etiology, CTP class, BCLC stage, ICI treatment, AFP | Empirical-unclear source, pneumonia, urinary tract infections, gastro-intestinal infections, nose and throat infections, encephalopaty, dental infections, skin infections, pre-TACE (transarterial chemoembolization) prophylaxis, blood borne/septicaemia | Beta-lactams, quinolones, cephalosporins, nitroimidazolem, rifampicin, macrolides, trimethoprim, glycopeptides, carbapenems, aminoglycosides, sulfonamides | Within 30 days before and after beginning ICIs | - |
| Spahn et al. | Univariable analysis | Systemic antibiotics, which included broad spectrum cephalosporins or fluoroquinolones (13); non-absorbable antibiotic rifaximin as a prophylaxis against hepatic encephalopathy (8). | | Within 30 days before and after beginning ICIs | - |
| Cheung et al. | Gender, age, ICI treatment, treatment history, alcohol-related diseases, dabetes mellitus, hypertension, ischemic heart disease, atrial fibrillation, congestive heart failure, stroke, hepatic decompensation, cause of cirrhosis, prednisolone, aspirin, statins, PPIs, prior sorafenib/lenvatinib use, AFP, bilirubin, alkaline phosphatase, alanine aminotransferase, aspartate aminotransferase, albumin, globulin, international normalized ratio, platelet, creatinine, sodium. | Fever of unknown cause or empirical use (35), pneumonia (7), gastrointestinal bleeding (5), post-TACE (transarterial chemoembolization) fever (5), biliary sepsis (4), rupture HCC (3), cellulitis (3), urinary tract infection (3), diarrhoea (2), hepatic encephalopathy (2), liver abscess (2), spontaneous bacterial peritonitis (1), thrombosed haemorrhoids (1), post-SIRT (selective internal radiation therapy) fever (1), transurethral resection of prostate (1), salmonella bacteremia (1), post-operative bile leak (1) | Penicillins (83), cephaloporins (28), macrolides (3), carbapenems (24), quinolones (26), tetracyclines (2), aminoglycosides (4), nitroimidazoles (14), glycopeptides (3), septrin (3), nitrofurantoin (1), rifampicin (1), rifaximin (2) | Within 30 days before and after beginning ICIs | 13 |
| Alshammari et al. | Univariable analysis | - | - | Within 14 days before and 28 days after beginning ICIs | - |
| Chen_xj et al. | Gender, age, surgical history, vascular invasion, extrahepatic metastases, viral etiology, treatment line, ECOG, BCLC stage, AFP | Post-interventional fever and chills (9), fever (4), abdominal infections (2), upper respiratory tract infections (1), urinary tract infections (1), preventing leukopenia prevention of infections (1) | Piperacillin sodium (7), sulbactam and cefoperazone (3), levofloxacin (3), efotaxime sodium and sulbactam sodium (2), layangtoubaona (2), imipenem and cilastatin sodium (1) | Within 60 days before and after beginning ICIs | - |
| Chen_q et al. | Univariable analysis | pneumonia, upper respiratory tract infections, urinary tract infections, bloodstream infections, surgery, incomplete bowel obstruction, etc. | β-lactams, β-Lactamaseinhibitors, quinolones, etc. | Within 60 days before and 30 days after beginning ICIs | 3-14 |

CTP, Child-Turcotte Pugh; BCLC, Barcelona clinic liver cancer; ICI, immune checkpoint inhibitor; ECOG, Eastern Cooperative Oncology Group; AFP, alpha fetoprotein; PPI, proton pump inhibitors; OS, overall survival; PFS, progression-free survival; TACE, transcatheter arterial chemoembolization.
